# Supplementary material for: Factors Predicting successful treatment outcome with novel BPaLM/BPaL regimen in individuals with drug-resistant tuberculosis: Experience From Indonesia
Source: PLoS One. 2026 Apr 1;21(4):e0345702. doi: 10.1371/journal.pone.0345702 (PMC13042743; doi:10.1371/journal.pone.0345702)
Supplement: S1 Table — (DOCX) [file pone.0345702.s001.docx]

Supplementation Files: Treatment-Related Side Effects

| **Side Effects** | **Number (%)**  **N = 132** |
| --- | --- |
| Gastrointestinal manifestation | 123 (93,1) |
| Peripheral neuropathy | 40 (30,3%) |
| Headache, Vertigo | 21 (15,9) |
| Athralgia | 27 (20,4) |
| Skin reaction | 14 (10,6) |
| Hearing impairment | 4 (3,0) |
