# Supplementary material for: Factors Predicting successful treatment outcome with novel BPaLM/BPaL regimen in individuals with drug-resistant tuberculosis: Experience From Indonesia
Source: PLoS One. 2026 Apr 1;21(4):e0345702. doi: 10.1371/journal.pone.0345702 (PMC13042743; doi:10.1371/journal.pone.0345702)
Supplement: Attachment — Submitted filename: Response to Reviewer.docx [file pone.0345702.s003.docx]

To:

**Graeme Hoddinott, Ph.D**

Plos One Academic Editor

Thank you for the constructive feedback on our manuscript. We appreciate the reviewers' positive assessment of the merit and relevance of our work, as well as your specific guidance regarding the "Clinical Implementation and Programmatic Success Factors" section. We have carefully addressed all the comments raised by the reviewers and made the necessary revisions to improve the manuscript.

Thank you again for the opportunity to revise and improve our manuscript. We believe these changes have substantially enhanced its scientific quality and clarity.

I am looking forward to hearing your positive response.

**Sincerely,**

Purwiga, MD

Corresponding Author

On behalf of all authors

**Editor Comments Section**

Thank you for your submission. As noted by the reviewers, it has significant merit and relevance.

In addition to the points raised by the reviewers, please also consider whether the section on "**Clinical implementation and programmatic success factors**" may instead be **better placed** **in the background section**. At current, it reads as your reflections on why there is such high success with BPAL / BPALM in your setting. But what **is the justification for your belief** that **these are the four reasons?** **To avoid the speculative component**, it would be stronger to describe **HOW the new regimens were implemented in the background / context**. And include some of the reflections as a much shorter, more circumspect set of propositions for further investigation in the discussion

We agree that the current placement and tone of this section require revision. Following your recommendations, we have made the following changes:

1. We have created a new subsection titled "BPaLM/BPaL Program Implementation Context" in the Methods section. The content is now presented as objective program description rather than reflective interpretation **(Line 218-238)**
2. We have significantly condensed the discussion of programmatic factors to brief paragraphs, stating: Present operational achievements (e.g., median time to treatment initiation) as descriptive findings and explicitly state that "the independent contribution of these programmatic elements to treatment success requires further investigation". Conclude with specific, testable propositions for future research **(Line 327-355)**

**Response to Reviewers**

**Reviewer #1:**

This is an **impressive and very relevant piece** of operational research that offers valuable real-world insight into the use of the BPaLM/BPaL regimen for drug-resistant tuberculosis (DR-TB) in Indonesia. The work is both timely and significant, particularly in light of the country’s heavy DR-TB burden and the World Health Organization’s recent endorsement of this shorter, all-oral regimen.

The manuscript is **well written and logically organized**, with clear objectives and a sound methodological approach. The study population is appropriately chosen, and the statistical analysis—especially the use of multivariate logistic regression—has **been applied correctly** to identify predictors of treatment success.

The results are compelling: the 88.8% treatment success rate, along with the identification of **treatment adherence** and **nutritional improvement as major determinants**, provides strong, actionable evidence for national TB programs. The discussion thoughtfully connects these findings with existing global and Indonesian data, offering realistic suggestions for strengthening patient support and adherence monitoring. Ethical oversight and data transparency are also well addressed.

In summary, this is a solid and well-executed study that adds real value to the growing evidence base on the BPaLM/BPaL regimen. **It makes both a scientific and policy contribution** and will be of great interest to clinicians, researchers, and program managers involved in DR-TB control.

We are pleased that the reviewer recognizes the timeliness and relevance of this operational research, particularly in the context of Indonesia's significant DR-TB burden and the recent implementation of BPaLM/BPaL regimens in our national program. We also appreciate the acknowledgment of our methodological rigor, the clinical significance of our findings, and the practical implications for TB program management. Thank you.

**Reviewer #2:**

The manuscript is **well-structured and addresses a scientifically important topic**. Nonetheless**, a few areas would benefit from additional clarification** to further strengthen the presentation.

| 1 | The retrospective study design is appropriate for the research objectives; however, additional **details regarding the inclusion and exclusion criteria would strengthen the methodological transparency**. In particular, clarification is needed on how patients were allocated to the BPaL versus BPaLM regimen and **whether specific baseline characteristics—such as linezolid intolerance, HIV co-infection, or other clinical factors—influenced regimen selection**. Providing this information would enhance the reader’s understanding of potential selection bias and comparability between the treatment groups. |
| --- | --- |
|  | We appreciate this important comment regarding treatment allocation and potential selection bias. We have addressed this by adding two new sections, "Treatment Allocation and Regimen Selection" and "Inclusion and Exclusion Criteria," to the Methods. These sections now clearly describe the criteria for selecting BPaLM versus BPaL regimens in our study **(Line 119-130 & Line 132-152).** We hope that this addresses the reviewer's concern regarding clarity and reproducibility  Regarding the specific baseline characteristics mentioned:  **Linezolid intolerance:** No patients had documented linezolid intolerance at baseline, as all were linezolid-naïve  **HIV co-infection:** As described in the regimen selection criteria, the choice between BPaLM and BPaL regimens was not determined by HIV status. In our study, only 1 patient (0.6%) had HIV co-infection, reflecting the low HIV prevalence in our setting.  **Other clinical factors:** An important consideration in regimen selection was the exclusion of pregnant and breastfeeding women, as evidence for BPaLM/BPaL use in this population remains limited based on ongoing studies.  We acknowledge that the retrospective design and clinician-directed treatment allocation may introduce potential selection bias. We have added this limitation to the Discussion section and noted that comparative effectiveness studies with randomization would be needed to definitively address this concern. **(Line 403-407).** |
|  |  |
| 2 | The statistical **methods are described briefly**. **Please specify** **which tests were used** for comparisons and justify their selection to improve clarity and reproducibility |
|  | We have expanded the Statistical Analysis section to improve clarity and reproducibility. The revised section now specifies: independent t-test for normally distributed continuous variables and Mann-Whitney U test for non-normally distributed variables; chi-square test or Fisher's exact test (when expected cell counts <5) for categorical variables; and multivariate logistic regression with backward elimination for identifying independent predictors. Variables with p<0.25 in bivariate analysis were included in the multivariate model **(Line 182-204).** |
|  |  |
| 3 | The manuscript **reports adverse events but provides limited detail**. Consider including **a table summarizing the frequency and severity of key events** (e.g., neuropathy, anemia, hepatotoxicity). |
|  | Thank you for this constructive suggestion. We have included Table 4, which summarizes treatment-related side effects observed during BPaLM/BPaL treatment in our cohort (N = 132 patients with documented adverse events). The table presents the frequency of key adverse events, including gastrointestinal manifestations (93.1%), peripheral neuropathy (30.3%), headache/vertigo (15.9%), arthralgia (20.4%), skin reactions (10.6%), and hearing impairment (3.0%).  We acknowledge that our adverse event documentation focused on clinically significant events that were systematically monitored during monthly follow-up visits as part of our standard DR-TB program protocol. While we documented the occurrence and frequency of these adverse events, detailed severity grading for each event was not consistently recorded in the medical records, as clinical management decisions were individualized by our multidisciplinary team based on overall patient assessment rather than standardized severity scoring systems. |
| 4 | In the Discussion, **please expand on operational challenges, such as drug supply, patient adherence, and monitoring, and discuss how these factors may impact the scale-up of the BPaL/BPaLM regimen within the national TB program** |
|  | We appreciate this valuable suggestion to discuss operational challenges relevant to national program implementation. We have added a new section in the Discussion titled "Operational Challenges and Program Implementation" that addresses: drug supply, patient adherence support, monitoring, and scale-up implications **(Line 327-355)**. |
